# A Calpain-Like Protein Is Involved in the Execution Phase of Programmed Cell Death of *Entamoeba histolytica*

**DOI:** 10.3389/fcimb.2018.00339

**Published:** 2018-09-25

**Authors:** Tania Domínguez-Fernández, Mario Alberto Rodríguez, Virginia Sánchez Monroy, Consuelo Gómez García, Olivia Medel, David Guillermo Pérez Ishiwara

**Affiliations:** ^1^Departamento de Infectómica y Patogénesis Molecular, CINVESTAV, Ciudad de México, Mexico; ^2^Programa de Biomedicina Molecular, Escuela Nacional de Medicina y Homeopatía (ENMyH), Instituto Politécnico Nacional, Ciudad de México, Mexico

**Keywords:** *Entamoeba histolytica*, cysteine proteases, programmed cell death, protein overexpression, calcium binding sites, calpain

## Abstract

Oxygen or nitrogen oxidative species and chemical stress induce the programmed cell death (PCD) of *Entamoeba histolytica* trophozoites. PCD caused by the aminoglycoside G418 is reduced by incubation with the cysteine protease inhibitor E-64; however, no typical caspases or metacaspases have been detected in this parasite. Calpain, a cysteine protease activated by calcium, has been suggested to be part of a specific PCD pathway in this parasite because the specific calpain inhibitor Z-Leu-Leu-Leu-al diminishes the PCD of trophozoites. Here, we predicted the hypothetical 3D structure of a calpain-like protein of *E. histolytica* and produced specific antibodies against it. We detected the protein in the cytoplasm and near the nucleus. Its expression gradually increased during incubation with G418, with the highest level after 9 h of treatment. In addition, a specific calpain-like siRNA sequence reduced the cell death rate by 65%. All these results support the hypothesis that the calpain-like protein is one of the proteases involved in the execution phase of PCD in *E. histolytica*. The hypothetical interactome of the calpain-like protein suggests that it may activate or regulate other proteins that probably participate in PCD, including those with EF-hand domains or other calcium-binding sites.

## Introduction

Programmed cell death (PCD) plays crucial roles in a multitude of physiological processes, such as embryogenesis, aging, and maintenance of cell populations in tissues (Ameisen, 1996). It involves the activation of a group of calcium-dependent cysteine proteases called “caspases” and a complex cascade of events characterized by distinct morphological and biochemical changes (Elmore, 2007). In *Entamoeba histolytica*, PCD had been induced *in vitro* by nitric oxide (Ramos et al., 2007), hydrogen peroxide (Nandi et al., 2010) and the aminoglycoside G418 (Villalba et al., 2007). Amoeba PCD is characterized by some typical biochemical and morphological changes described in other organisms, such as a considerable increase in cytosolic calcium, a reduction in intracellular potassium, intracellular pH acidification and chromatin condensation (Villalba et al., 2007). The PCD phenotype diminish when trophozoites are incubated with E-64, a specific cysteine protease inhibitor; nevertheless, genes encoding for caspases have not been identified in *E. histolytica* (Villalba et al., 2007). Interestingly, Nandi et al. (2010) demonstrated that the activity of calpain-like increase during H_2_O_2_-induced PCD. Moreover, a specific inhibitor of calpain activity (Z-Leu-Leu-Leu-al) decrease DNA fragmentation and increase cellular viability of trophozoites during G418-induced PCD (Sanchez-Monroy et al., 2015).

Calpains, a group of non-lysosomal Ca^2+^-dependent cysteine proteases, have been identified in almost all eukaryotes and bacteria, but not in archaebacteria. Among the 15 members of the calpain family found in human, the ubiquitous calpains 1 and 2 are the most intensely studied. Calpains have been involved in various physiological processes such as cell proliferation, cell cycle progression (Glading et al., 2002), signal transduction (Carafoli and Molinari, 1998), cell migration, cytoskeletal remodeling (Zhang et al., 2011) and in the regulation of cell death (Squìer et al., 1994; Tagliarino et al., 2001; Harwood et al., 2005). In fact, calpain was the first protease identified in initiating apoptosis (Squìer et al., 1994). Several studies have notably highlighted how closely these proteases are linked to caspases. Calpains 1 and 2 cleave several members of caspase family, activating the caspase-3,−7, and−12 and inactivating the caspase-8 and -9 (Chua et al., 2000; Nakagawa and Yuan, 2000). By regulating caspases, calpains can thus control indirectly apoptosis. Also, in situations of mass calcium influx, membrane transection or ischemia/reperfusion injury, the ubiquitous calpains are activated and in turn trigger caspase-3 (Wang, 2000).

In addition to the typical morphological events related to nuclear and membrane changes (Kerr et al., 1972), apoptosis accompanies a dramatic reorganization of the cytoskeleton due to the selective proteolysis of vital cellular substrates. Thus, regulated proteolysis by calpain is required for the control of fundamental cellular processes including cytoskeletal remodeling, and activation of proteolytical cascades leading to apoptosis (Saido et al., 1994).

Calpains are heterodimeric proteins, consisting of two subunits of 80 and 28 kDa (Croall and Ersfeld, 2007). The large subunit in classical calpains consists of four conserved domains: An N-terminal anchor helix (Nter), a catalytic protease core domain (CysPc) with the two subdomains PC1 and PC2, a C2-like domain (C2L), and a penta-EF-hand domain (PEF). Non-classical calpains lack both the Nter and the PEF domain and may contain additional domains in combination with CysPc (Hosfield et al., 1999; Strobl et al., 2000; Joyce et al., 2012).

Atypical or unconventional calpains are described as calpain-like proteins that contain only a CysPc consensus signature with variations in the catalytic triad and no PEF-containing domain is present, and they may also contain additional domains in combination with CysPc (Sorimachi et al., 2010). Calpain-like proteins have mainly been found in invertebrates and lower eukaryotes. In Trypanosoma and *Blastocystis hominis*, calpain-like proteins have been involved in the life cycle, the differentiation process and the regulation of PCD (Hertz-Fowler et al., 2001; Giese et al., 2008; Yin et al., 2010). In *Entamoeba histolytica* upregulation of calpain-like gene very early during PCD induction, correlates with the release of cytosolic calcium (Villalba et al., 2007; Sanchez-Monroy et al., 2015) and calpain activity increased after 6 h of PCD induction (Sanchez-Monroy et al., 2015).

In this study, we modeled a hypothetical 3D structure of the calpain-like protein of *E. histolytica* based on the conserved domains previously identified in the primary protein sequence (Sanchez-Monroy et al., 2015). By Western blot (WB) and confocal microscopy analyses, we demonstrated that the expression of calpain-like protein increased during PCD induction, localizing the protein in the cytoplasm and near the nucleus. Knockdown of the calpain-like gene by a specific small interference RNA sequence (siRNA) provoked a 65% decrease in PCD. The results presented here support the hypothesis that the calpain-like protein plays an important role in the execution phase of *E. histolytica* PCD. In addition, a hypothetical interactome of the calpain-like protein suggests that other proteins, including some with calcium-binding domains, also participate in the PCD pathway of this parasite.

## Materials and methods

### *Entamoeba histolytica* culture

Trophozoites of clone A (Orozco et al., 1983), which is a virulent subclone of strain HM1: IMSS, were axenically cultured in TYI-S-33 medium at 37°C, harvested at a logarithmic growth phase, as described (Diamond et al., 1978). PCD was induced by exposure to 10 μg/ml G418 for different periods of time, as indicated.

### *In silico* analysis of the *E. histolytica* calpain-like protein

The sequence of the calpain-like protein from *E. histolytica* was obtained from NCBI (XP_657312.1) (EHI_045290). Then, a hypothetical 3D structure was predicted by the I-TASSER program (Protein Structure and Function Predictions). The UCSF CHIMERA program was used to compare the tertiary structure of the calpain-like from *E. histolytica* with the tertiary structure of the calpain-1, (mu/I) large subunit [*Homo sapiens*] (AAH08751) and the PyMOL software tools were used to visualize the preserved calpain-like domains (Sanchez-Monroy et al., 2015). The similarity of the proteins included in our study was compared with the available protein homologs against non-redundant databases like BLASTP program of NCBI.

### qRT-PCR assays

Total RNAs from trophozoites without treatment or treated with 10 μg/ml G418 for 0.5, 1.5, 3, 6, and 9 h were isolated using the Trizol reagent (Invitrogen) according to the manufacturer's protocol. cDNA was synthesized using an oligo(dT) primer and the Superscript II reverse transcriptase (Invitrogen). For qRT-PCR assays, specific primers for the *calpain-like* gene (XM_652220.1) were designed by the Primer Express Software for Real-Time PCR version 3.0 (Applied Biosystems), (sense primer: 5′-GTTTCAATATCACAACCT CGTTGTG-3′ and antisense primer: 5′-AAAGTCTCTCCAGAATCACCTCCA-3′). As an internal control, we used specific primers for the *gapdh* gene (XM_651327.2, XM_645264.2 and XM_649267.2) (sense 5′-CCGTCCACAGACAATTCGAA-3′; antisense 5′TTGAGCTGGATCTCTTTCAGCTT-3′ primers). Reactions were performed in the ABI PRISM 7000 Sequence Detection System (Applied Biosystems) by monitoring the real-time increase in fluorescence using the SYBR Green PCR Master Mix (Applied Biosystems). The relative quantification was calculated using the CT method, which uses the formula 2^−ΔΔ*CT*^ (Livak and Schmittgen, 2001). Statistically significant differences in gene expression between non-induced and PCD-induced trophozoites were analyzed by comparisons of the means of three independent biological replicates in triplicate using the Tukey's test with GraphPad Prism statistical software version 6.0.

### Production of calpain-like antibodies

Antigenic peptides from calpain-like protein were analyzed by the ABCpred program (Saha and Raghava, 2006). Peptides with higher antigenic scores were used as probes for BLAST search of the *E. histolytica* genome. The CCEWKGKWRDDDPAWT polypeptide, situated at positions 230–246, was synthesized coupled to the KLH (Keyhole Limpet Hemocyanin) tag to increase its immunogenicity (GL Biochem). Four BALB/c mice were immunized by the subcutaneous and intramuscular routes using 50 μg of the polypeptide emulsified in Titer-Max Gold adjuvant (1:1) (Sigma). Then, the animals were immunized with two more doses (100 μg) of the polypeptide resuspended in the adjuvant at 15-day intervals followed by bleeding to obtain antibodies. The pre-immune serum was obtained before the first immunization.

The experimental protocol was approved by the institutional committee for animal care and provided all technical specifications for the production, care and use of laboratory animals (NOM-062-ZOO-1999).

### Western blot assays

Trophozoites (2 × 10^7^) without treatment or treated with 10 μg/ml G418 for 0.5, 1.5, 3, 6, and 9 h were harvested and washed twice with cold PBS. For the extraction of total proteins, cells were hypotonically lysed for 20 min at 4°C in the presence of a mixture of protease and phosphatase inhibitors (PMSF, 1 mM; leupeptin, 10 μM; N-ethylmaleimide, 25 mM; PHMB, 2.5 mM; E-64, 5 μM; Na_3_VO_4_, 1 mM; NaF, 50 mM; iodoacetamide, 5 mM) and 1X Complete Protease inhibitor (Roche). Proteins were separated by 12% sodium dodecyl sulfate polyacrylamide gel electrophoresis (SDS-PAGE), transferred to nitrocellulose membranes, and non-specific binding sites were blocked with 5% fat-free milk. Membranes were incubated with calpain-like (dilution 1:3,500) or GAPDH (dilution 1:5,000, Santa Cruz Biotechnology) antibodies. Afterwards, membranes were incubated with a secondary HRP-labeled antibody (Invitrogen). Finally, protein bands were visualized by chemiluminescence (ECL, GE Healthcare, United Kingdom), and the MicroChemi system (Biostep). Pre-immune serum was used as a control.

### Immunofluorescence and confocal microscopy

After treatments in test tubes (brand Pyrex), trophozoites (1 × 10^6^) were placed on cover slides for approximately 20 min, fixed and permeabilized with 100% methanol for 5 min. Non-specific binding sites were blocked with 1% horse serum for 1 h at 37°C. Cell slides were incubated overnight at 4°C with α-calpain-like antibodies (1:40), rinsed with PBS three times, incubated with anti-mouse secondary antibody conjugated to Alexa 488 (1:400) for 1 h at room temperature and rinsed again three times with PBS. Nuclei were stained with 4′,6-diamidino-2-phenylindole (DAPI), and samples were observed in a confocal microscope (Carl Zeiss LSM 700) using the ZEN 2009 software. About 10 amoebas were chosen at random from each slide, fluorescence of each amoeba represents the intensity mean value of 25 optical sections from the top to the bottom of each cell. The images were representative of three independent experiments.

### Knockdown of the calpain-like gene

The complete mRNA sequence (XM_652220.1) of the calpain-like gene was analyzed by the online program Target finder (https://www.genscript.com/tools/sirna-target-finder) to obtain potential small interference RNA (siRNA) sequences, which were evaluated by a nucleotide BLAST on NCBI (https://blast.ncbi.nlm.nih.gov). A specific siRNA sequence of the *calpain-like mRNA* corresponding to nucleotides 6-31 (sense: 5′- UACUGACGAUGAAUUUCCAGCUGA-3′; antisense: 5′- UUCAGCUGGAAAUUCAUCGUCAGUA−3′) was synthesized (Ambion). As a negative control, an additional siRNA sequence of non-related sequence (NRS) using the following set of sense and antisense primers was synthesized (sense: 5′-CAAGCUGACCCUGAAGUUCdTdT−3′; antisense: 5′-GAACUUCAGGGUCAGCUUGdTdT-3′).

The uptake of siRNAs by trophozoites was carried out by the soaking method as previously described (Ocádiz-Ruiz et al., 2013). Briefly, trophozoites (1 × 10^6^) collected from 90% confluent cultures were inoculated in 25-ml culture plastic flasks (Corning) containing TYI-S-33 medium and incubated at 37°C for 24 h. Then, the double-stranded siRNAs calpain-like or NRS sequences (50 nM) were added to the cultures and incubated at 37°C for 24 h. To confirm the knockdown of the calpain-like gene, qRT-PCR and WB assays were performed as described above.

### Terminal deoxynucleotidyltransferase-mediated dUTP nick-end labeling (TUNEL) assays

Trophozoites were fixed in 4% formaldehyde for 2 h at 4°C. After the samples were washed twice with PBS, 50 μl of TUNEL reaction mixture (Roche) was added and incubated for 60 min at 37°C in a humidified atmosphere in the dark. Then, trophozoites were rinsed four times with PBS, and nuclei were counterstained with DAPI and mounted with VECTASHIELD (Vector Laboratories). The samples were observed through a confocal microscope (Carl Zeiss LSM 700) using the ZEN 2009 software. As a positive control, trophozoites were treated with 20 mg/ml DNase I endonuclease (Invitrogen) for 30 min, and non-treated trophozoites were used as a negative control.

### Flow cytometry

To determine the effect of calpain-like gene silencing on the viability of trophozoites subjected to PCD, cells were stained with propidium iodide (PI) and analyzed by flow cytometry. Briefly, after 24 h of treatment with the siRNA, trophozoites were incubated for 9 h with G418; then, cells were suspended in 1 ml of PBS and incubated for 5 min on ice in the dark with 1 mg/ml PI (Invitrogen). Finally, fluorescence was examined in a BD LSRFortessa cell analyser (BD Biosciences).

### Bioinformatic analysis

The STRING 10.5 protein-protein interaction database (https://string-db.org/) (Szklarczyk et al., 2015) was used to determine the potentially involved known and predicted protein networks. For these analyses, we used a medium confidence value and non-filtered disconnected nodes.

### Statistical analysis

Statistically significant differences in WB, immunofluorescence and trophozoites viability were analyzed by comparisons of the means of three independent biological replicates by ANOVA using the GraphPad Prism statistical software version 6.0.

## Results

### Identification of conserved domains in the protein architecture and 3D structure of calpain-like

**Figure 1:**
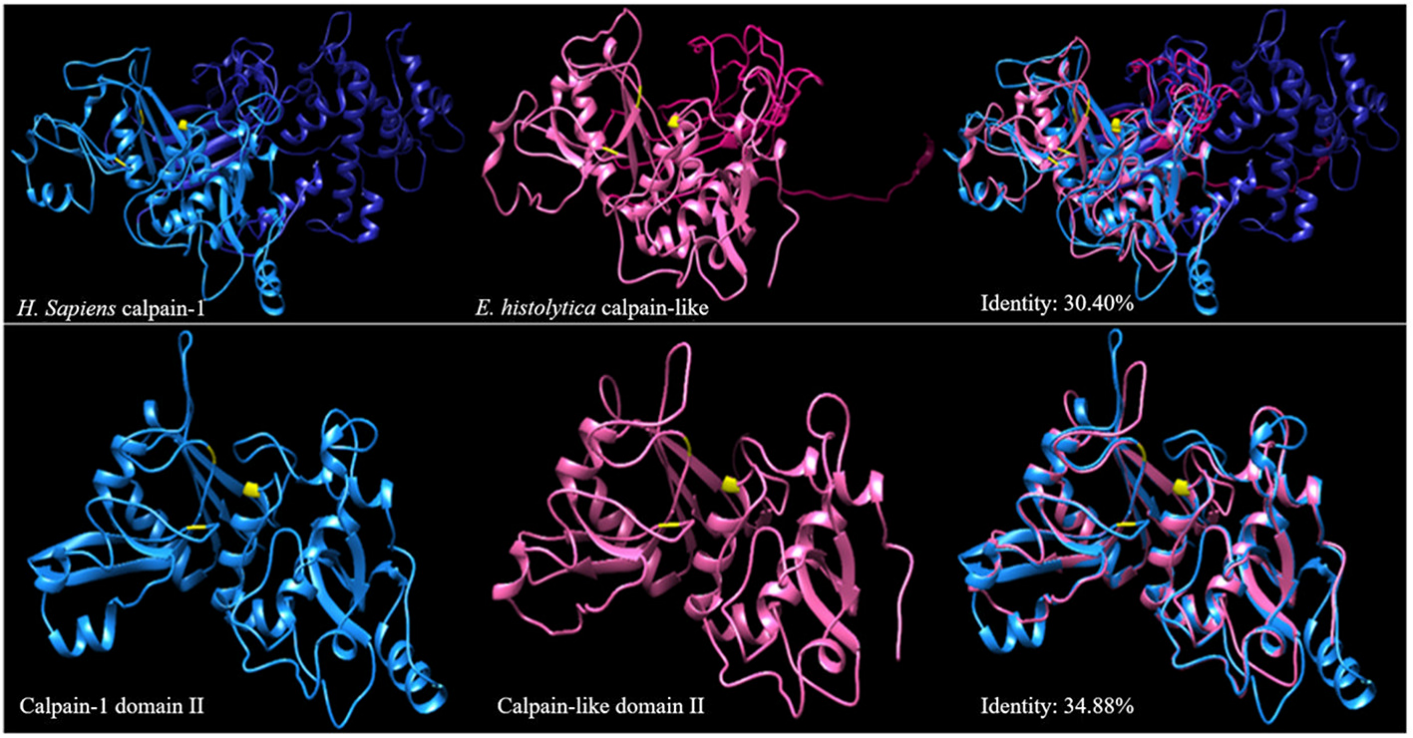
3D structures of the calpain-like protein of *E. histolytica* and human calpain-1. The diagram represents the 3D structures of human calpain (blue) and the calpain-like protein of *E. histolytica* (pink). Upper panels show the 3D models of the complete calpains. Lower show the 3D structures of the domain II of both calpains. Right shows the overlapping in the 3D calpain structures, the numbers indicate the percentage of identity. The amino acids that make up the catalytic triad of calpain are represented in yellow.

The I-TASSER program database was used to predict the three-dimensional molecular structure of calpain-like (XP_657312.1) from *E. histolytica* (Figure 1, pink structure) and human (Figure 1, blue structure) calpain-1 protein obtained from NCBI (AAH08751). One of the templates used by I-TASSER to predict both structures was a catalytic domain of human calpain-1 crystal (PDB ID: 2ARY). Using the UCSF CHIMERA, we obtained a 30.4% identity between the complete proteins. Furthermore, the 3D structure of catalytic domain of human calpain-1 (the domain II that contain amino acid residues that form the catalytic triad were identified and indicated in yellow by the PyMOL software tools) showed a higher identity (34.88%) (Figure 1). The BLASTP analysis of calpain-1 (mu/I) large subunit [Homo sapiens] yielded 33% identity with calpain-like of *E. histolytica*.

### Expression and location of calpain-like protein within trophozoites

**Figure 2:**
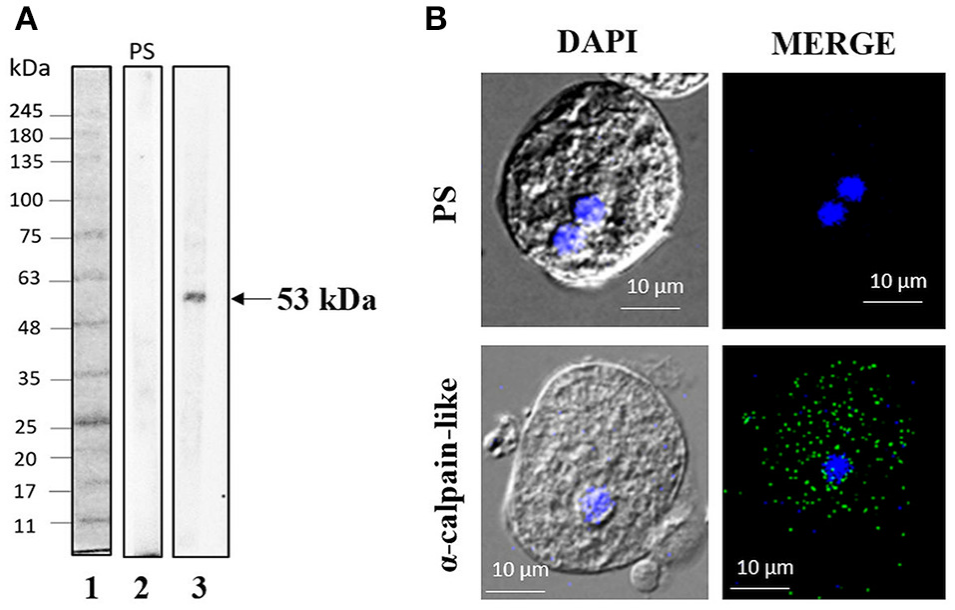
Expression and localization of the calpain-like protein in trophozoites. **(A)** Total extracts of *E. histolytica* were separated by 12% SDS-PAGE and analyzed by WB assays using pre-immune serum (PS, 2) or mouse α -calpain-like antibody (3). FPL-007 Flash protein ladder molecular marker (Gel company) (1). **(B)** Representative image of laser confocal microscopy of methanol-fixed trophozoites using mouse α-calpain-like antibody. PS, pre-immune serum.

By performing WB analysis on trophozoite extracts using specific antibodies against calpain-like protein, we detected a single 53-kDa band, whereas the pre-immune serum did not recognize any band (Figure 2A). Immunofluorescence assays using the same antibodies showed that the calpain-like protein is located throughout the cytoplasm in non-treated trophozoites (parasites cultured in TYI-S-33 medium, without G418) (Figure 2B).

### Expression of the calpain-like protein during PCD induced by G418

**Figure 3:**
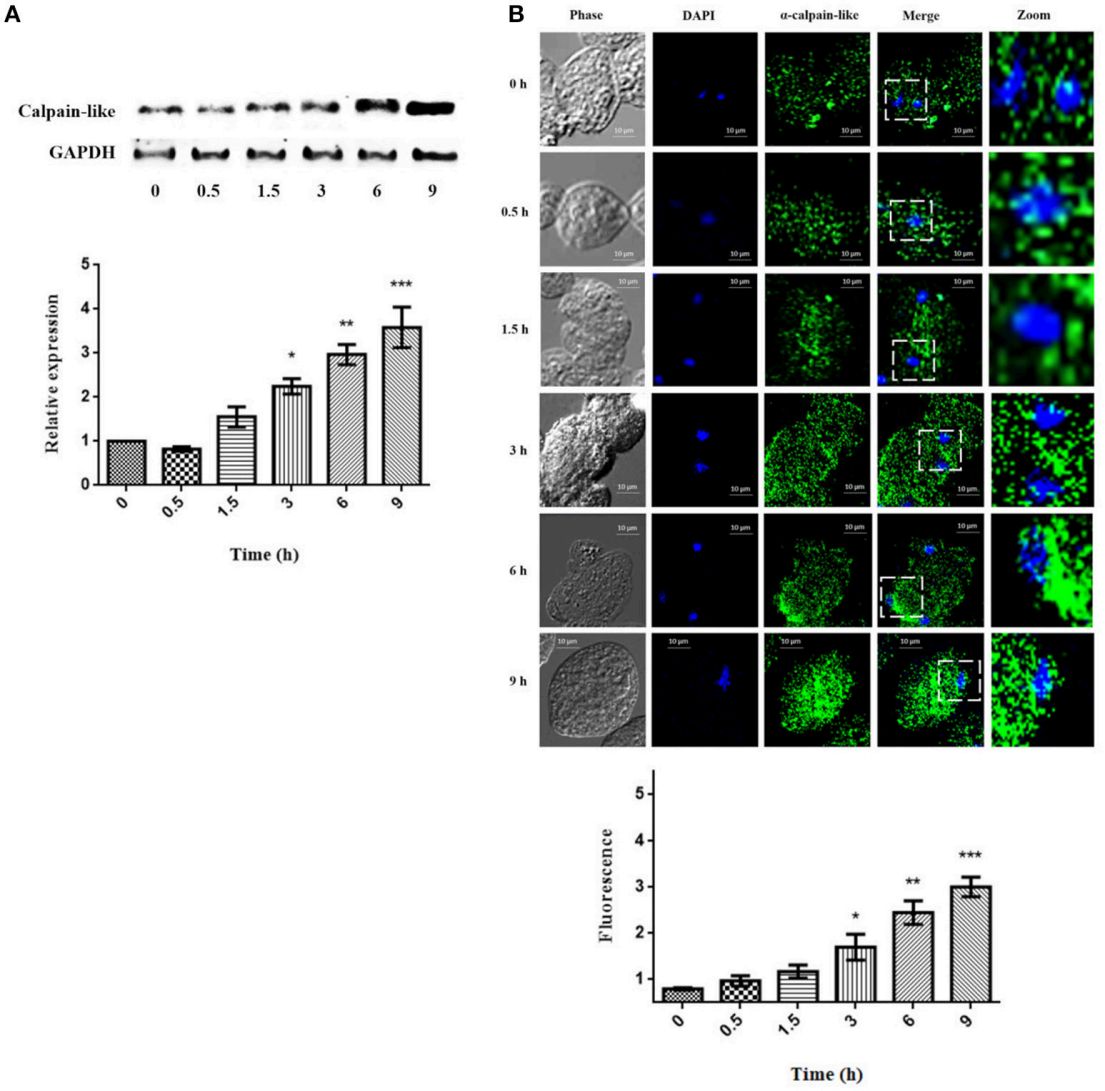
Expression of the calpain-like protein during PCD induced by G418. **(A)** Expression of the calpain-like protein in non-treated trophozoites (0 h) and treated with 10 μg/ml of G418 for 0.5, 1.5, 3, 6, and 9 h. The graph shows the densitometry analysis of calpain-like protein expression levels. The α-GAPDH reactivity was used as an internal loading control for normalization of experimental results. Asterisks indicate times in which calpain-like relative expression showed statistically significant difference *p < 0.05, **p < 0.01, ***p < 0.001. **(B)** Distribution of calpain-like protein during PCD. Trophozoites were incubated with G418 at 37°C for the indicated times. Then, samples were prepared for laser confocal microscopy and stained with mouse α-calpain-like antibody and the secondary antibody. Phase, phase contrast images; DAPI, nucleus; Merge, co-localization between DAPI and calpain-like protein. The graph shows the densitometry analysis of calpain-like protein fluorescence. Asterisks indicate times in which calpain-like fluorescence showed statistically significant difference *p < 0.05, **p < 0.01, ***p < 0.001.

Calpain-like protein expression increased slightly in a time-dependent manner (1.7-, 2.3-, 3.0-, and 3.5-fold) after 1.5, 3, 6, and 9 h of PCD induction by G418, respectively (Figure 3A). On the other hand, the distribution and location of the calpain-like protein in trophozoites during the PCD process was almost the same as in un-treated trophozoites, but the signal was increased, with the maximum expression at 9 h. Interestingly, at 6 and 9 h, the calpain-like protein was also re-located closer to the nucleus (Figure 3B).

### Silencing of the calpain-like gene

**Figure 4:**
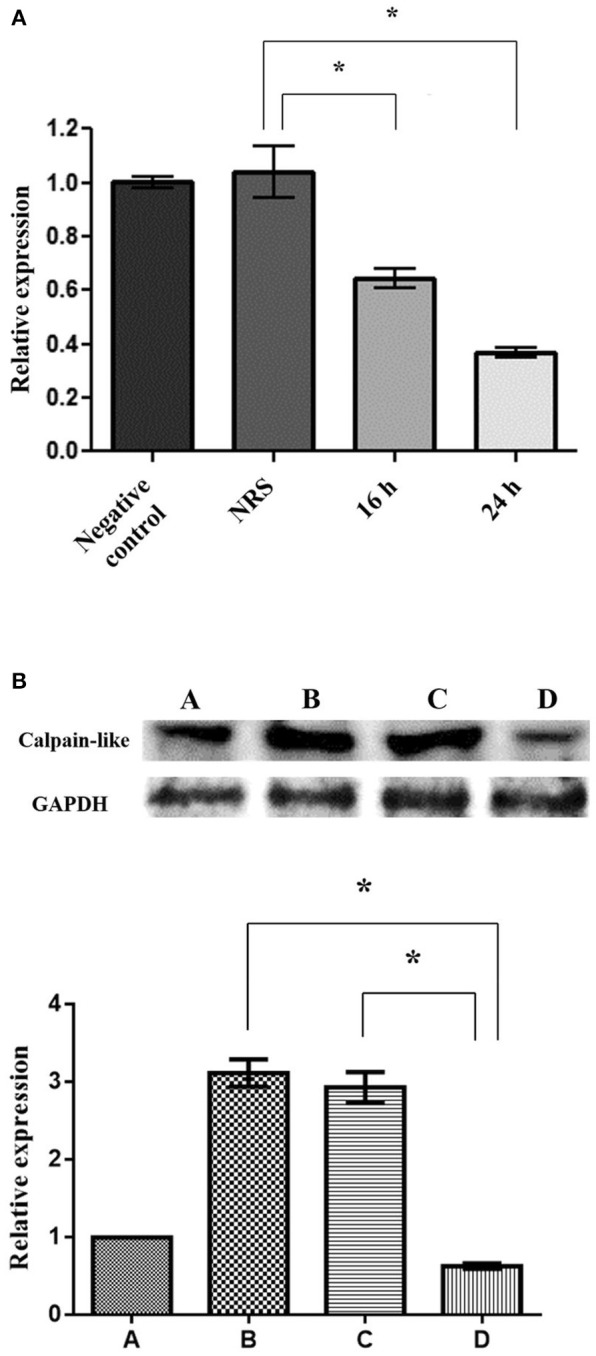
Silencing of the calpain-like gene. **(A)** qRT-PCR expression of calpain-like gene from trophozoites incubated with 50 μg/ml of the calpain-like siRNA sequence for 16 and 24 h. As control, trophozoites were incubated with a non-related sequence (NRS). Negative control represents trophozoites without siRNA incubation. **(B)** WB of calpain-like protein. (A) Negative control, untreated trophozoites; (B) Trophozoites after a 9-h incubation with 10 μg/ml of G418; (C,D) Trophozoites pre-incubated for 24 h with NRS (C) or calpain-like (D) siRNAs sequences and incubated 9 h with 10 μg/ml G418. The graph shows the densitometry analysis of calpain-like protein expression levels. *indicate statistically significant difference (*P* < 0.05).

To evaluate the role of the calpain-like protein in the execution phase of PCD, we silenced its gene expression through a small interference RNA (siRNA) sequence. First, using qRT-PCR, we analyzed the effect of specific calpain-like silencing with 50 nM/ml siRNAs for 16 and 24 h. The results showed 37 and 67% decrement of the calpain-like gene expression after 16 and 24 h, respectively (Figure 4A). As expected, incubation with NRS siRNA sequence for the same time (24 h) did not affect calpain-like gene expression (Figure 4A). By WB we observed that calpain-like protein expression increased 3.0- and 3.5-fold times in control trophozoites induced to PCD or in trophozoites treated with NRS siRNA sequence, respectively, in comparison to the basal expression of control trophozoites. Interestingly, PCD induced trophozoites treated with calpain-like siRNA sequence significantly reduced (80%) the calpain-like protein expression (Figure 4B).

### Effect of calpain-like silencing on PCD

**Figure 5:**
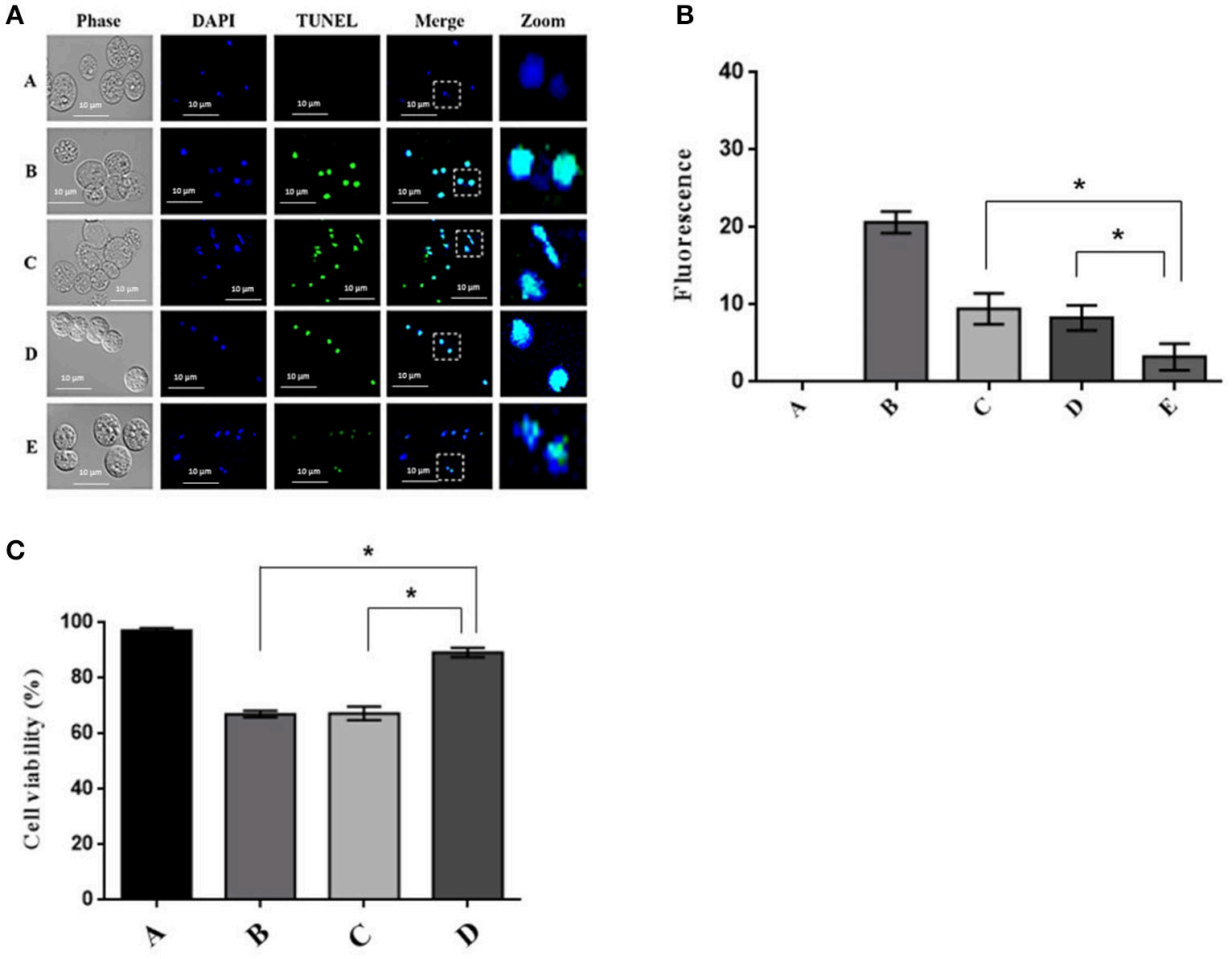
Effect of calpain-like silencing during PCD. **(A)** DNA fragmentation in calpain-like silenced trophozoites after PCD induction by G418. Confocal microscopy analysis of trophozoites showing the TUNEL staining, samples were counter-stained with DAPI. (A) Negative control, untreated trophozoites; (B) Positive control, trophozoites treated with 20 μg of DNase I endonuclease for 30 min; (C) Trophozoites after a 9-h incubation with 10 μg/ml of G418; (D,E) Trophozoites pre-incubated for 24 h with NRS (D) or calpain-like (E) siRNAs sequences and then 9 h with 10 μg/ml G418; **(B)** Densitometric analysis of (A). **(C)** Effect of calpain-like silencing on the cell viability of *E. histolytica* incubated with G418; (A) Negative control, untreated trophozoites; (B) Trophozoites after a 9-h incubation with 10 μg/ml of G418; (C,D) Trophozoites pre-incubated for 24 h with NRS (C) or calpain-like (D) siRNAs sequences and then 9 h with 10 μg/ml G418. *indicate statistically significant difference: *P* < 0.05.

The trophozoites incubated with the siRNA for 24 h and subsequently with G418 for 9 h were analyzed by TUNEL assay (*In Situ* Cell Death Detection Kit, AP). The results showed that silencing of the calpain-like gene caused a considerable decrease in positive tunnel staining compared to treatment with G418 alone for 9 h or NRS treatment for 24 h and PCD induction for 9 h (Figure 5A). Densitometric analysis of three independent experiments, showed that calpain silencing trophozoites displayed a significant reduction of fluorescence (65%) in comparison to PCD induced trophozoites or those treated with NRS siRNA sequence (Figure 5B). Consistently, silencing the calpain-like gene expression increased 90% cell viability of trophozoites after 9-h PCD induction, while trophozoites treated only with G418 for 9 h or treated with NRS followed by a 9-h PCD induction, cell viability was approximately 63% (Figure 5C).

### Searching for putative protein interaction networks of calpain-like protein

**Figure 6:**
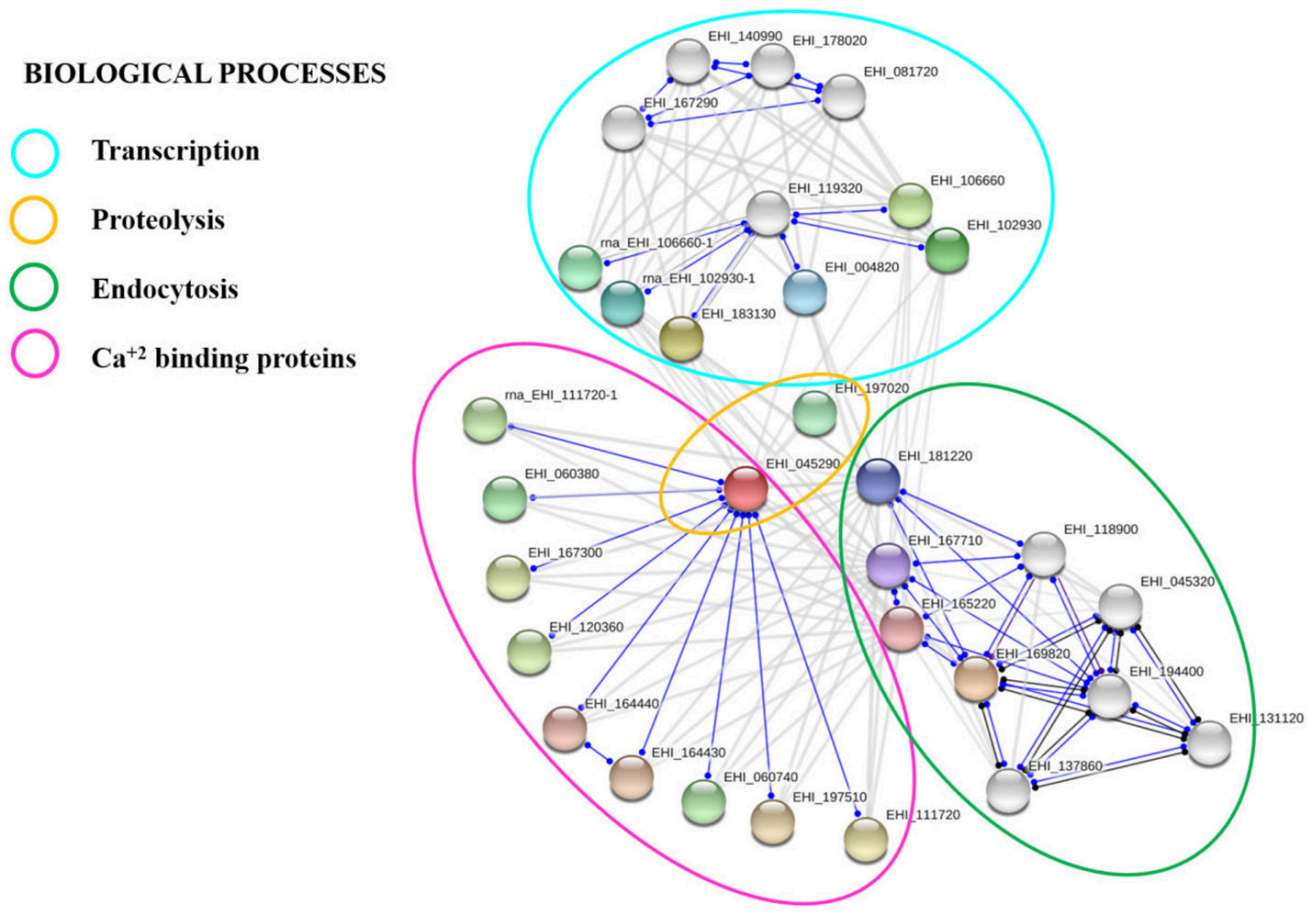
Putative protein interaction networks. STRING 10.5 protein-protein interaction program (https://string-db.org) was used to predict biological processes as well as interactive networks where the calpain-like protein may be involved.

We analyzed the calpain-like protein in the STRING 10.5 database to create a putative network of proteins that interact with it. To make the prediction, we selected the interactome based on text mining, experiments, databases, co-expression, neighborhood, gene fusion, and co-occurrence. The results suggested that 20 proteins could have several possible interactions among them with a *p*-value of 1.23e-07 (Figure 6). This interactome contains proteins related to endocytosis, such as vesicular trafficking proteins, proteins related to vacuolar transport, proteins with zinc-finger domains related to DNA binding, and proteins with EF-hand calcium binding domains, such as grainins 1 and 2 (Table 1).

**Table 1:** Component proteins of the predicted interactome of the calpain-like protein.

**ID**	**Name**	**Funtion**
**TRANSCRIPTION**		
EHI_140990	Leucine rich repeat / protein phosphatase 2C domain containing protein	•Metal ion binding •Phosphoprotein phosphatase activity
EHI_167290	Leucine rich repeat / protein phosphatase 2C domain containing protein	•Metal ion binding •Phosphoprotein phosphatase activity
EHI_081720	Leucine rich repeat / protein phosphatase 2C domain containing protein	•Maintenance of epithelial cell apical/basal polarity •Catalytic activity •Signal transduction
EHI_178020	Leucine rich repeat / protein phosphatase 2C domain containing protein	•Catalytic activity
EHI_106660	Putative uncharacterized protein	•Nucleic acid binding
EHI_102930	Putative uncharacterized protein	•Nucleic acid binding
EHI_119320	Histone deacetylase	•Hydrolysis of an N_6_-acetyl-lysine residue of a histone to yield a deacetylated histone
EHI_004820	Putative uncharacterized protein	•Nucleic acid binding
EHI_183130	Putative uncharacterized protein	•Nucleic acid binding
rna_EHI_102930-1	Putative uncharacterized protein	•Nucleic acid binding
rna_EHI_106660-1	Putative uncharacterized protein	•Nucleic acid binding
**PROTEOLYSIS**		
EHI_197020	Signal peptidase, putative	•Cleavage of hydrophobic, N-terminal signal or leader sequences from secreted and periplasmic proteins. •Signal peptide processing
EHI_045290	Calpain family cysteine protease, putative	•calcium-dependent cysteine-type endopeptidase activity. •Metal ion binding
**Ca**^+2^ **BINDING PROTEINS**		
rna_ EHI_111720-1	Grainin 2	•Calcium ion binding
EHI_060380	Grainin, putative	•Calcium ion binding
EHI_167300	Grainin 1	•Calcium ion binding
EHI_120360	Grainin, putative	•Calcium ion binding
EHI_164440	Actinin-like protein, putative	•Calcium ion binding
EHI_164430	Actinin-like protein, putative	•Calcium ion binding
EHI_060740	EF-hand calcium-binding domain containing protein	•Calcium ion binding •Metal ion binding
EHI_197510	EF-hand calcium-binding domain containing protein	•Calcium ion binding
EHI_111720	Grainin 2	•Calcium ion binding
**ENDOCYTOSIS**		
EHI_181220	Adhesin; Adhesin 112 (EhADH112)	•Endosome transport via multivesicular body sorting pathway
EHI_167710	Putative uncharacterized protein	•Uncharacterized protein
EHI_165220	Putative uncharacterized protein	•Uncharacterized protein
EHI_118900	Vacuolar sorting protein VPS4, putative	•ATP binding
EHI_045320	Vacuolar protein sorting 36, putative	•Ubiquitin binding •Protein transport to vacuole involved in ubiquitin-dependent protein catabolic process via the multivesicular body sorting pathway
EHI_169820	SNF7 family protein	•Vacuolar transport
EHI_194400	SNF7 family protein	•Vacuolar transport
EHI_137860	Putative uncharacterized protein	•protein homodimerization activity •Protein transport to vacuole involved in ubiquitin-dependent protein catabolic process via the multivesicular body sorting pathway
EHI_131120	ELL complex EAP30 subunit, putative	•Protein transport to vacuole involved in ubiquitin-dependent protein catabolic process via the multivesicular body sorting pathway

## Discussion

Programmed cell death involves a complex cascade of events characterized by distinct morphological and biochemical changes triggered by a group of cysteine proteases (Samali et al., 1999). In eukaryotes, the expression and activation of cysteine proteases such as caspases, metacaspases, or calpain occur when the cytosolic Ca^2+^ concentration increases (Elmore, 2007). Calpains, enzymes belonging to the family of calcium-dependent cysteine proteases, have also been implicated in pro-apoptotic pathways by the cleavage of apoptosis-associated proteins, such as caspase 7 (Gafni et al., 2009), Bax, Bcl-2 (Gao and Dou, 2000), Jun and Fos (Hirai et al., 1991), caspase 12 and Jnk (Tan et al., 2006). Several findings in higher eukaryotes have suggested a role of calpains in the execution phase of PCD. For instance, an inhibitor of calpains has been reported to prevent apoptosis of glial cells induced by silibinin, a natural polyphenolic flavonoid (Jeong et al., 2011). In addition, calpain-2 has been demonstrated to play a crucial role in hydrogen peroxide-induced apoptosis in pancreatic AR42J cells (Hiemer et al., 2017).

*E*. *histolytica* has no canonical caspases. However, PCD is inhibited by E-64, a specific inhibitor of cysteine proteases (Villalba et al., 2007), and calpain-like activity increases when trophozoites are induced to PCD by nitric oxide species (Nandi et al., 2010). Typical calpains contain four structural domains: domain I, which is cleaved after Ca^2+^ activation; domain II, which contains the active site (the catalytic triad of cysteine, asparagine and histidine) conserved throughout the family and residues that can bind two Ca^2+^ atoms implicated in the enzymatic activation; domain III, which contains a phospholipid-binding motif in the area of C2 (Clan 2 within the classification of the cysteine protease family); and domain IV, which contains five EF-hand motifs that bind Ca^2+^ (Smith and Schnellmann, 2012). Interestingly, typical caspases have not been identified in *E. histolytica*, but a caspase-like enzyme has been suggested to be involved in PCD (Sanchez-Monroy et al., 2015). In this work, we predicted the 3D structure of the calpain-like protein of *E. histolytica*, and within this structure, we located domain II, containing the catalytic triad residues (Cys57, His206, and Asn226). Despite being phylogenetically distant from typical calpain, domain II of the calpain-like protein of *E. histolytica* shows a 34.88% identity with the same domain of human calpain-1.

Using a specific antibody against the calpain-like protein, we detected a 53-kDa band that displayed a time-dependent induction during PCD, obtaining a maximum expression after 9 h of incubation with G418. This fact is in concordance with the enzymatic activity, which showed the highest calpain activity at longer times of PCD induction (Sanchez-Monroy et al., 2015).

Subcellular localization of calpain-like protein by confocal microscopy showed that this enzyme was distributed diffusely in the cytoplasm of non-treated trophozoites, but after PCD induction, in addition to its higher expression, it apparently relocated closer to the nucleus.

Regulated proteolysis by calpain is required for the control of fundamental cellular processes including cytoskeletal remodeling, membrane fusion, cell proliferation and differentiation, and activation of proteolytical cascades leading to apoptosis (Saido et al., 1994). This fact is in concordance with our findings that calpain-like protein is expressed basally in the cytoplasm. On the other hand, when trophozoites are exposed to G418 calpain-like protein also increased, probably due to its participation in the cytoskeleton rearrangement, during apoptosis. Once activated, calpains degrade membrane, cytoplasmic and nuclear substrates, leading to the breakdown of cellular architecture (Momeni, 2011).

In higher eukaryotes, calpains have been suggested to be involved in DNA fragmentation via endonuclease activation (Squier and Cohen, 1997; Villa et al., 1998), and as effector proteases that cleave cellular proteins involved in DNA repair. For instance, upon activation, human μ-calpain is translocated to the nucleus, where it cleaves PARP and p53 (Tagliarino et al., 2001). On the other hand, calpain located in the nucleus of *Plasmodium falciparum* has been associated with the progression of the cell cycle (Russo et al., 2009). Thus, the observation of the calpain-like protein in the peri-nuclear area of trophozoites 9 h after PCD induction suggests that this protease may participate in the downstream activation of proteins related to DNA fragmentation. This hypothesis is supported by an increase in trophozoite viability and by the blockage of DNA fragmentation in parasites treated with the specific calpain inhibitor Z-Leu-Leu-Leu-al during PCD induction (Sanchez-Monroy et al., 2015).

Here, we showed that knockdown of the calpain-like protein diminished DNA fragmentation and increased cell viability of trophozoites incubated for 9 h with G418, supporting the hypothesis that this cysteine protease participates in the execution phase of PCD. Similarly, the siRNA-mediated knockdown of calpain-1 in neuronal cultures submitted to apoptosis prevented the translocation of the apoptosis-inducing factor (AIF) to the nucleus, thus increasing cell viability (Cao et al., 2007; Jeong et al., 2011), and the knockdown of calpain-2 increased the viability of pancreatic AR42J cells treated with hydrogen peroxide (Hiemer et al., 2017).

Protein-protein interaction experiments currently in progress will allow us to identify other proteins that participate in the PCD of this parasite. We initiated this study by analyzing the putative interactome using the String 10.5 database. The results suggest that at least 20 proteins may have various possible interactions among them with a *p*-value of 1.23e-07. Interactions of these proteins with the calpain-like protein have not yet been reported in the *E. histolytica* database. We identified proteins that participate in endocytosis, such as vps36 and snf7, zinc binding proteins, as well as proteins that can bind calcium, including the grainins 1 and 2, which contain EF-hand calcium binding domains. Interestingly, previous studies have demonstrated an increase in the expression of grainins 1 and 2 at 30 min of PCD induction with G418, suggesting that these proteins could act as negative regulators of this event attempting to regulate the cytosolic concentration of free calcium related to the activation of PCD (Monroy et al., 2010).

This assumption is because other proteins with EF-hands, such as calmodulins (CaM) and calmodulin-dependent protein kinase (CaMKIV), that interact with calpain are critical for improved survival. In addition to CaM, other Ca^2+^-binding proteins, including calpains, play important roles in signal transduction leading to the control of cell proliferation as well as cell death. *In vitro*, Ca^2+^ is known to change calpain conformation, affecting its autocatalytic cleavage and activation (Strobl et al., 2000). Additionally, the Ca^2+^-activated protease calpain has been shown to be a pro-apoptotic factor by its ability to cleave and activate various proteins implicated in the apoptotic process (Momeni, 2011). Interestingly, virtually all proteins that bind to CaM are also calpain substrates (Wang et al., 1989); thus, an increase in intracellular Ca^+2^ stimulates the breakdown of proteins necessary for proliferation and viability and triggers programmed cell death (Berridge et al., 2003; Smyth and Putney, 2012).

Other proteins such as the androgen receptor (AR), which is required for growth in both androgen-sensitive and androgen-insensitive prostate cancer are cleaved by calpain in the presence of high Ca^2+^ concentrations due to the release of calpain from the calpain-CaM-calpastatin complex. In addition, the calpain-mediated proteolysis of CaMKIV has been demonstrated to trigger apoptosis in cultured neurons (Tremper-Wells and Vallano, 2005).

Considering an update classification of cell death focusing on mechanistic and essential morphological, biochemical, and functional aspects of the process (Galluzzi and Vitale, 2018), we should expeculate that amoeba PCD could be related to the lysosome-dependent cell death (LDCD). This type of cell death is induced by similar stimulus such as perturbations of intracellular homeostasis, cellular stress, ROS (reactive oxygen species) and Ca^2+^ imbalance. LDCD is demarcated by the permeabilization of lysosomal membranes and the release of lysosomal contents, including proteolytic enzymes of the cathepsin family, to the cytoplasm (Aits and Jäättelä, 2013). Other triggers include lysosomotropic agents (e.g., sphingosine), and the activation of calpains (Gómez-Sintes et al., 2016). ROS play a prominent causal role in LMP (lysosomal membrane permeabilization), not only as the H_2_O_2_-driven luminal production of hydroxyl radicals (Kurz et al., 2008), but also as ROS favor the activation of lysosomal Ca^2+^ channels (Sumoza-Toledo and Penner, 2011). Overall, the results presented here provide strong evidence that the calpain-like protein plays an important role in the execution phase of PCD in *E. histolytica*. We identified here *in silico*, some proteins that could interact with calpain-like of *E. histolytica*. Molecular and biochemical experiments currently in progress will allow us to discover new insights about the lysosomal or other pathways roles in controlling *E. histolytica* PCD.

## Author contributions

TD-F conceived and carried out experiments, analyzed data and drafted the manuscript. MR and CG participated in the design of the study, analyzed data and drafted the manuscript. VS and OM collaborated in the qRT-PCR and WB assays. DPI conceived and designed the study, analyzed data and drafted the manuscript.

### Conflict of interest statement

The authors declare that the research was conducted in the absence of any commercial or financial relationships that could be construed as a potential conflict of interest.

## Abbreviations

PCD: programmed cell death
PBS: phosphate-buffered saline
WB: Western blot
GAPDH: glyceraldehyde-3-phosphate dehydrogenase
NRS: non-related sequence
CaM: calmodulin
CaMKIV: calcium/calmodulin-dependent protein kinase IV
AR: androgen receptor
LDCD: lysosome-dependent cell death
ROS: reactive oxygen specieS
LMP: lysosomal membrane permeabilization.
